# *C. elegans* Ring Finger Protein RNF-113 Is Involved in Interstrand DNA Crosslink Repair and Interacts with a RAD51C Homolog

**DOI:** 10.1371/journal.pone.0060071

**Published:** 2013-03-28

**Authors:** Hyojin Lee, Arno F. Alpi, Mi So Park, Ann Rose, Hyeon-Sook Koo

**Affiliations:** 1 Department of Biochemistry, College of Life Science & Biotechnology, Yonsei University, Seoul, Republic of Korea; 2 Scottish Institute for Cell Signaling, University of Dundee, Dundee, United Kingdom; 3 Department of Medical Genetics, University of British Columbia, Vancouver, Canada; Florida International University, United States of America

## Abstract

The Fanconi anemia (FA) pathway recognizes interstrand DNA crosslinks (ICLs) and contributes to their conversion into double-strand DNA breaks, which can be repaired by homologous recombination. Seven orthologs of the 15 proteins associated with Fanconi anemia are functionally conserved in the model organism *C. elegans*. Here we report that RNF-113, a ubiquitin ligase, is required for RAD-51 focus formation after inducing ICLs in *C. elegans*. However, the formation of foci of RPA-1 or FCD-2/FANCD2 in the FA pathway was not affected by depletion of RNF-113. Nevertheless, the RPA-1 foci formed did not disappear with time in the depleted worms, implying serious defects in ICL repair. As a result, RNF-113 depletion increased embryonic lethality after ICL treatment in wild-type worms, but it did not increase the ICL-induced lethality of *rfs-1/rad51C* mutants. In addition, the persistence of RPA-1 foci was suppressed in doubly-deficient *rnf-113;rfs-1* worms, suggesting that there is an epistatic interaction between the two genes. These results lead us to suggest that RNF-113 and RFS-1 interact to promote the displacement of RPA-1 by RAD-51 on single-stranded DNA derived from ICLs.

## Introduction

Fanconi anemia (FA) is a rare recessive disease involving bone marrow failure, developmental abnormalities including short stature and rudimentary (or absent) thumbs, and susceptibility to cancers [1], [2]. The cells of FA patients have chromosomal defects including breaks, gaps, and rearrangements, and are especially hypersensitive to interstrand DNA crosslinking agents. There are 15 complementation groups of FA and the corresponding proteins have all been identified. The 15 FA-associated proteins include FANC A, B, C, D1, D2, E, F, G, I, J, L, M, N, O, and P, eight of which (A, B, C, E, F, G, L, and M) form a core complex. FANCM in the core complex is the first protein that recognizes interstrand DNA crosslinks (ICLs), and FANCL of the complex is an E3 ligase that mono-ubiquitinates FANCD2 [1], [3]–[6]. The mono-ubiquitination of FANCD2 is a key step in the FA pathway, leading to nuclear focus formation of FANCD2, and the mono-ubiquitin of FANCD2 becomes bound to FAN1 nuclease and SLX4 [5], [7]–[9]. FANCD2 acts as a switch in repair of double-strand DNA breaks (DSBs), guiding DSBs to homologous recombination rather than to nonhomologous end-joining [10], [11]. The newest members of the FA family are FANCN/PALB2, a binding partner of BRCA2, which functions in homologous recombination repair [12]–[14], FANCO/RAD51C, which recruits RAD51 to ICL sites [15], [16], and FANCP/SLX4, which forms a platform for nucleases [17]–[19]. Besides the 15 FA proteins, FA-associated proteins such as FAAP24, FAAP100, and FAAP20 participate actively in ICL repair [20]–[24].

In the model organism *C. elegans*, 7 of the 15 FA proteins (D1/BRC-2, D2/FCD-2, I/FNCI-1, J/DOG-1, M/FNCM-1, O/RFS-1, P/HIM-18) have been identified and their roles in ICL repair have been confirmed for all except the FANCP homolog [25]–[35]. Thus, seven proteins of the FA core and FANCN still remain to be identified in *C. elegans*. One possibility is that in this simple organism the core complex is made up of fewer components, given that only FANCM and FANCL of the core complex have been shown to have well-defined functions in mammalian cells. On the other hand, it is possible that the amino acid sequences of these core complex components are not conserved to levels recognizable by homology searches. Thus, we have taken a functional approach to identifying candidate FA components.

The FANCD2 homolog, FCD-2, has been reported to be mono-ubiquitinated or associated with a ubiquitinated protein [26], [27]. However, the E3 ligase has not been found in *C. elegans*. A ring finger protein, RNF-113, was shown to interact with FCD-2 in a high throughput yeast two-hybrid assay [36]–[38]. RNF-113 was predicted to be an E3 ligase and a candidate for the enzyme that ubiquitinates FCD-2 [32]. In this study, we have investigated the function of RNF-113 in response to ICL-induction using proliferating germ cells of *C. elegans*, and characterized its genetic relationships to FCD-2/FANCD2, RFS-1/RAD51C, and RAD-51.

## Results

### RNF-113 has a role in preventing DNA crosslink hypersensitivity

**Figure 1:**
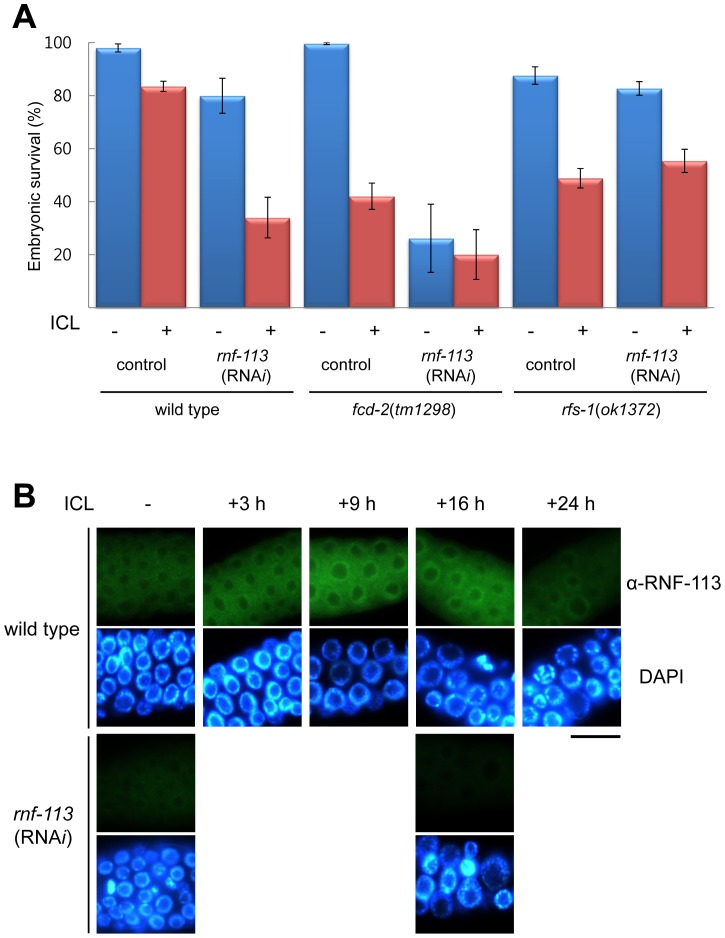
Effects of RNF-113 depletion on *C. elegans* survival after interstrand DNA crosslinking (ICL) and the intracellular localization of RNF-113. (A) Comparison of embryonic hatching rates after knocking down RNF-113 expression with and without ICL treatment. *rnf-113* RNA*i* was performed from the P0 young adult stage by feeding wild-type *C. elegans* worms *E. coli* cells expressing double-strand RNA for *rnf-113*. F1 worms at the L4 stage were treated with TMP (25 µg/ml) plus UVA, and eggs were collected over 24 h and their hatching scored 20 h later. This set of experiments in the wild-type background, was also performed in *fcd-2(tm298)* and *rfs-1(ok1372)* mutants. The error bars are SEM (standard errors of the mean). (B) Intracellular localization of RNF-113 in the germ cells of the mitotically proliferating region of *C. elegans* gonads was detected using polyclonal antibodies against RNF-113. Immuno-staining was performed at 3, 9, 16, and 24 h after ICL treatment in the wild type. The scale bar is 10 µm.

In a high throughput yeast two-hybrid assay a *C. elegans* ring finger protein, RNF-113, interacted with a number of proteins including DAF-16 (FOXO homolog) and FCD-2 (FANCD2 homolog) [36], [37]. The *C. elegans* protein is most closely related (35% identity in amino acid sequence) to human RNF113A (Figure S1 in File S1), a ring finger protein for which no biological function has been reported. Although *C. elegans* RNF-113 does not have significant sequence identity with mammalian FANCL, its physical interaction with FCD-2 suggested a possible role for this prospective ubiquitin ligase in modifying FCD-2. Therefore, we knocked down RNF-113 (see Materials and Methods) and examined the effect on repair of interstrand DNA crosslinks (ICLs). The RNA*i*-treated worms without exogenously-induced DNA damages produced embryos, only 80(±7 SEM)% of which hatched later (Figure 1A), suggesting a role of RNF-113 in normal embryogenesis. We then investigated the response of *rnf-113* knockdown worms to treatment with the crosslinking agent, TMP (4,5′,8-trimethylpsoralen), followed by exposure to UVA radiation. The photoactivated bifunctional psoralen induces DNA crosslinks, which occur almost exclusively between DNA strands, resulting in a very low level of intrastrand-crosslinks [39]. The ICL-treated L4 worms produced 33(±8)% hatched embryos after *rnf-113* knockdown, which was much lower than the corresponding value of 83(±2)% in wild-type worms. The yield (33±8%) of *rnf-113*(RNA*i*) worms after ICL treatment was thus much lower than the yield (63±7%) obtained by simply taking into account the embryonic lethality (17±2%) induced by ICLs in wild-type worms and the death (20±7%) resulting from *rnf-113* knockdown. The synergism between ICL treatment and *rnf-113* knockdown leads to the conclusion that RNF-113 plays a critical role in repairing DNA crosslinks.

To see whether RNF-113 interacts genetically with FCD-2, we depleted RNF-113 in the *fcd-2(tm1298)* mutant. These depleted worms laid only 12 embryos, compared with 241 from the wild type, 201 from *fcd-2(tm1298)*, and 100 from *rnf-113*(RNA*i*) (Figure S2A in File S1), and the percentage of hatched embryos from the doubly deficient worms (26±12%) was much lower than from the untreated *rnf-113* (80±7%) worms. In fact, the number of progeny of the doubly deficient worms was too small to measure an effect of ICL on survival (Figures 1A and S2A in File S1). To investigate the reasons for this very small brood size, we examined the germ line, and found that the number of endomitotic (Emo) oocytes in the *fcd-2(tm1298)*;*rnf-113*(RNA*i*) worms was much higher than in either singly-deficient strains (Figure S2B in File S1). The ‘Emo’ phenotype was previously observed in oocytes with defective ovulation and resulted from uncontrolled DNA replication [40]. The endomitotic oocytes observed in our study contained either conglomerate chromosomes with enormous amount of DNA or greatly increased numbers of bivalent condensed chromosomes (Figure S2B in File S1).

### The nuclear level of RNF-113 increases in response to ICLs

To probe the intracellular location of RNF-113, we stained the gonads of wild-type and *rnf-113*(RNA*i*) worms with RNF-113 antibody (Figure 1B). The protein was present in the cytoplasm and on the periphery of the nuclei of germ cells. After ICL treatment, the amount of protein increased in both nucleus and cytoplasm, the maximum effect being seen between 9 h and 16 h after treatment i.e. the period when the germ cells were enlarged due to cell cycle arrest. The accumulation of RNF-113 decreased over time and had almost disappeared by 24 h in most of the germ cells. This corresponds to the time when cell cycling resumed based on nuclear size (Fig. 1B) and the disappearance of RAD-51 foci (data not shown). The specificity of the antibody was confirmed using RNA*i* knockdown of RNF-113, which effectively eliminated the anti-RNF-113 signal. The result also confirmed the efficiency of the RNA*i* treatment.

### RNF-113 is required not for FCD-2 focus formation but for RAD-51 focus formation after ICL treatment

**Figure 2:**
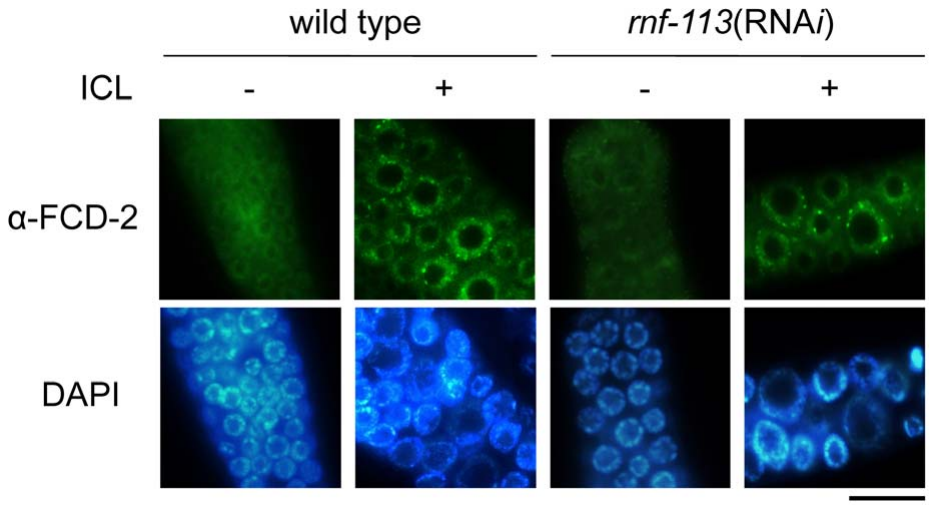
Focus formation by FCD-2 after ICL treatment is not affected by RNF-113 depletion. The mitotically proliferating regions of wild-type and *rnf-113*(RNA*i*) gonads were immuno-stained using FCD-2 polyclonal antibody at 18 h after ICL (TMP/UVA) treatment. The scale bar is 10 µm.

Since RNF-113 interacted with FCD-2 in a high throughput yeast two-hybrid assay [36]–[38], we examined the effect of its depletion on FCD-2 focus formation. FCD-2 (FANCD2 homolog) appeared as nuclear foci in wild-type germ cells 18 h after ICL, as previously reported, and the FCD-2 foci formed even upon the depletion of RNF-113 (Figure 2) [26], [33]. Most of the FCD-2 foci disappeared by 24 h after ICL in both of wild-type and RNF-113 depleted cells (data not shown). In mammalian cells, FANCD2 is ubiquitinated by FANCL, and as a result forms nuclear foci [3], [5]. Therefore, the fact that FCD-2 focus formation is not affected by *rnf-113* knockdown suggests that FCD-2 is not a ubiquitination target of RNF-113.

**Figure 3:**
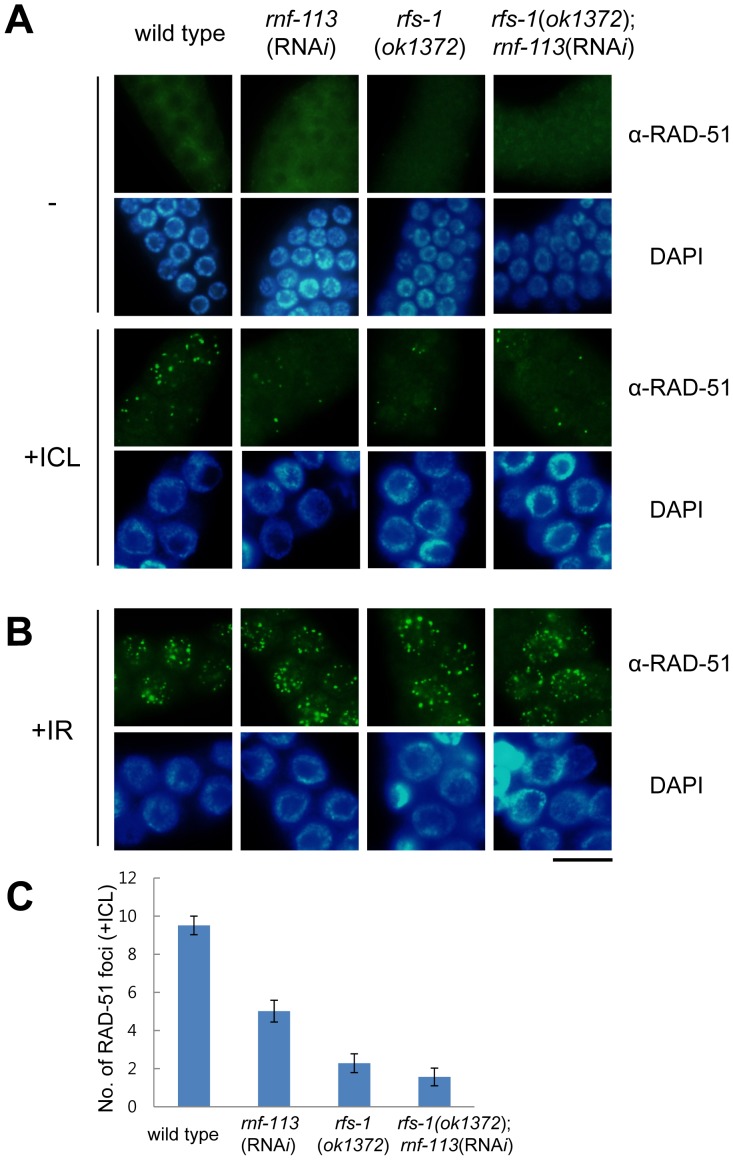
RNF-113 depletion attenuates RAD-51 focus formation at ICLs in wild-type worms, but not in *rfs-1* mutants. (A) The mitotically proliferating regions of gonads from wild-type, *rnf-113*(RNA*i*), *rfs-1(ok1372)*, and *rfs-1(ok1372);rnf-113*(RNA*i*) worms are shown after staining for RAD-51 at 16 h following TMP/UVA treatment. (B) The immuno-staining in (A) was repeated at 3 h after IR (ionizing radiation, 75 Gy) treatment instead of ICL treatment. The scale bars are 10 µm. (C) A focal plane having a maximum number of RAD-51 foci was chosen for each nucleus of germ cells (n = 150 for wild-type and *rnf-113*(RNA*i*); n = 30 for *rfs-1(ok1372)* and *rfs-1(ok1372);rnf-113*(RNA*i*)) 16 h after ICL treatment as in (A), and the average numbers of RAD-51 foci per nuclear focal plane are plotted. The error bars are SEM.

Having observed that FCD-2 focus formation was not affected by RNF-113 knockdown, we examined RAD-51 focus formation in germ cell nuclei [41]. In mammalian cells, RAD51, acting downstream of FANCD2, is recruited to DSBs resulting from incisions at ICLs and initiates repair of the DSBs via homologous recombination [42], [43]. As observed previously [33], RAD-51 formed nuclear foci after ICL treatment in wild-type *C. elegans* germ cells, the focus signal being most prominent between 9 h and 18 h (Figures 3A and S3A in File S1). However, when RNF-113 was depleted, the number of RAD-51 foci was greatly reduced (from 9.5±0.5 (SEM) foci to 5.0±0.6 foci per nuclear focal plane), and the number did not increase significantly with time after ICL treatment (Figures 3 and S3A in File S1). This indicates that RNF-113 is needed for efficient loading of RAD-51 on DNA sites damaged by interstrand DNA crosslinking.

Because RAD-51 focus formation after ICL treatment was greatly diminished by RNF-113 depletion, we asked if the level of RAD-51 protein or its activation by phosphorylation was affected by RNF-113 depletion (Figure S4A in File S1). *C. elegans* worm extracts were separated on a 10% SDS-polyacrylamide gel, and RNA-51 was probed by western blotting. Two bands of RAD-51 were seen in wild-type worms before and after ICL treatment. The intensity of the upper band increased after the ICL treatment, while that of the lower band decreased. This shift to the upper band is thought to be due to phosphorylation of RAD-51, as mammalian RAD51 is phosphorylated by CHK1 after DNA damage [44]. RNF-113 knockdown did not significantly reduce either the level of RAD-51 protein or affect its gel mobility shift, indicating that RNF-113 is not needed for phosphorylation of RAD-51. As a control, we observed that *chk-1* RNA*i* partly inhibited the shift to the upper band, supporting the conclusion that the upper band is a phosphorylated form of RAD-51. We conclude that RNF-113 regulates focus formation by RAD-51 at ICLs without affecting the amount or the phosphorylation of RAD-51.

### RNF-113 is not required for RAD-51 focus formation after DSB formation

In order to examine whether RNF-113 responds specifically to ICLs and not to other types of DNA damage such as DSBs (double-strand DNA breaks), we probed RAD-51 focus formation after irradiating worms with γ-rays. RAD-51 foci appeared clearly at 3 and 9 h after γ-irradiation in both wild-type and *rnf-113*(RNA*i*) germ cells (Figures 3B and S3B in File S1), suggesting that RNF-113 is required for repair of ICLs, but not for DSBs induced by γ-rays. In agreement with these results, RNF-113 depletion did not increase embryonic lethality after γ-irradiation (Figure S5B in File S1). Nevertheless, RNF-113 protein increased in the germ cells after the irradiation (Figure S5A in File S1), leaving open the possibility that RNF-113 plays a minor role in DSB repair downstream of RAD-51.

### Loss of RFS-1 has an epistatic relationship with RNF-113 deficiency in terms of ICL-sensitivity and RAD-51 focus formation

RNF-113 depletion resulted in hypersensitivity to ICL-inducing agents and greatly attenuated the resulting RAD-51 focus formation. To further characterize this effect, we examined RFS-1, a RAD51C homolog, which is required for RAD-51 focus formation after ICL treatment but not after ionizing-radiation [28]. For this purpose, we measured embryonic lethality in the deletion mutant *rfs-1(ok1372)* with or without depletion of RNF-113 (Figure 1A). The *rfs-1* mutant showed an embryonic survival of 49(±4)% in the presence of ICL-inducing agents, much lower than for the wild type (83±2%), in agreement with a previous report [28]. Depletion of RNF-113 in the *rfs-1* mutant did not increase embryonic lethality either with or without ICL treatment (p values 0.54 and 0.55, respectively). In contrast, embryonic survival after RNF-113 depletion in the wild-type strain was 80(±7)% before ICL treatment and 34(±8)% after the treatment. The fact that the doubly-deficient *rfs-1*;*rnf-113*(RNA*i*) strain was not significantly more sensitive to ICLs than the singly defective *rnf-113*(RNA*i*) strain (Student's *t* test, *p* value = 0.12) or the *rfs-1* strain (*p* = 0.55), demonstrates that RNF-113 functions in the same pathway as RFS-1. The *rfs-1* mutation also reversed the decreased brood size of *rnf-113*(RNA*i*) worms seen in the absence of exogenous ICLs (Figure S2A in File S1).

Since the *rfs-1* mutation was epistatic to *rnf-113* knockdown in terms of the survival of embryos derived from germ cells exposed to ICL agents (Figure 1A), we tested whether the two genes also interacted in the same way with respect to RAD-51 focus formation (Figures 3A and 3C). In agreement with the report by Ward et al. [28], the number of RAD-51 foci after ICL treatment was reduced by *rfs-1(ok1372)* mutation to one fourth the level in the wild type (from 9.5±0.5(SEM) foci to 2.3±0.5 foci per nuclear focal plane). This reduction was two-fold greater than that caused by *rnf-113* knockdown. In the doubly-deficient *rfs-1*;*rnf-113* (RNA*i*) strain, the number of RAD-51 foci did not decrease further (Student's *t* test, *p* = 0.30). This epistatic interaction between *rnf-113* and *rfs-1* in RAD-51 focus formation agrees well with the data on embryonic survival after ICL treatment (Figure 1A).

### ICL-induced RPA-1 foci do not disappear with time in RNF-113-depleted cells

**Figure 4:**
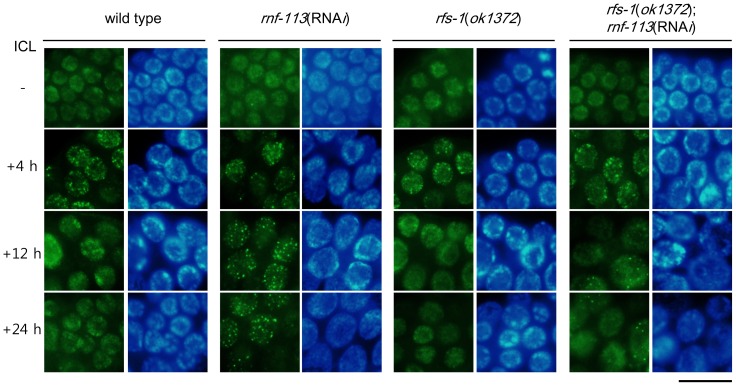
RNF-113 depletion does not affect the formation of RPA-1 foci after ICL treatment, but greatly retards the dissipation of RPA-1 foci. The mitotically proliferating regions of gonads from wild-type, *rnf-113*(RNA*i*), *rfs-1(ok1372)*, and *rfs-1(ok1372);rnf-113*(RNA*i*) worms are shown after immuno-staining for RPA-1 at 4 h, 12 h, and 24 h following TMP/UVA treatment. The scale bar is 10 µm.

RPA-1, which is the large subunit of RPA in *C. elegans*, activates the checkpoint pathway involving ATL-1 (ATR homolog) and CHK-1 in *C. elegans* [45], and is essential for the FCD-2 focus formation [33]. RPA-1 focus formation reached a maximum by 4 h (usually between 3 h and 6 h) after ICL formation in wild-type worms, and most of the foci had disappeared by 12 h, probably due to their replacement by RAD-51 (Fig. 4). RPA-1 focus formation was normal in RNF-113-depleted cells as measured at 4 h after ICL treatment (Figure 4). This was expected, since nuclear foci of FCD-2, which acts downstream of RPA-1, formed normally (Figure 2). RPA-1 foci even formed normally in *rfs-1(ok1372)* cells, since RFS-1 is only involved in the assembly and disassembly of RAD-51 filaments, not in earlier steps. The rate of dissipation of RPA-1 foci in *rfs-1(ok1372)* cells was also very similar to that in the wild type, although the formation of RAD-51 foci and ensuing homologous recombination were defective in these cells. In contrast to the situation of wild-type and *rfs-1(ok1372)* worms, RPA-1 foci did not disappear with time in the RNF-113-depleted worms, most of them being still present 24 h after ICL treatment. This suggests that the stalled replication forks or single-stranded DNA regions to which RPA-1 binds, persist in the depleted strain due to serious defects in ICL repair. In comparison, the *rfs-1*;*rnf-113*(RNA*i*) strain was similar to the *rfs-1* mutant with respect to the dynamics of RPA-1 foci, although a minor fraction of RPA-1 foci remained 24 h after ICL treatment. These results agree well with the data on embryonic survival in Figure 1A, showing that embryonic survival after ICL was not significantly decreased by *rnf-113* depletion in the *rfs-1* mutant background.

### RNF-113 has ubiquitin ligase activity in vitro

**Figure 5:**
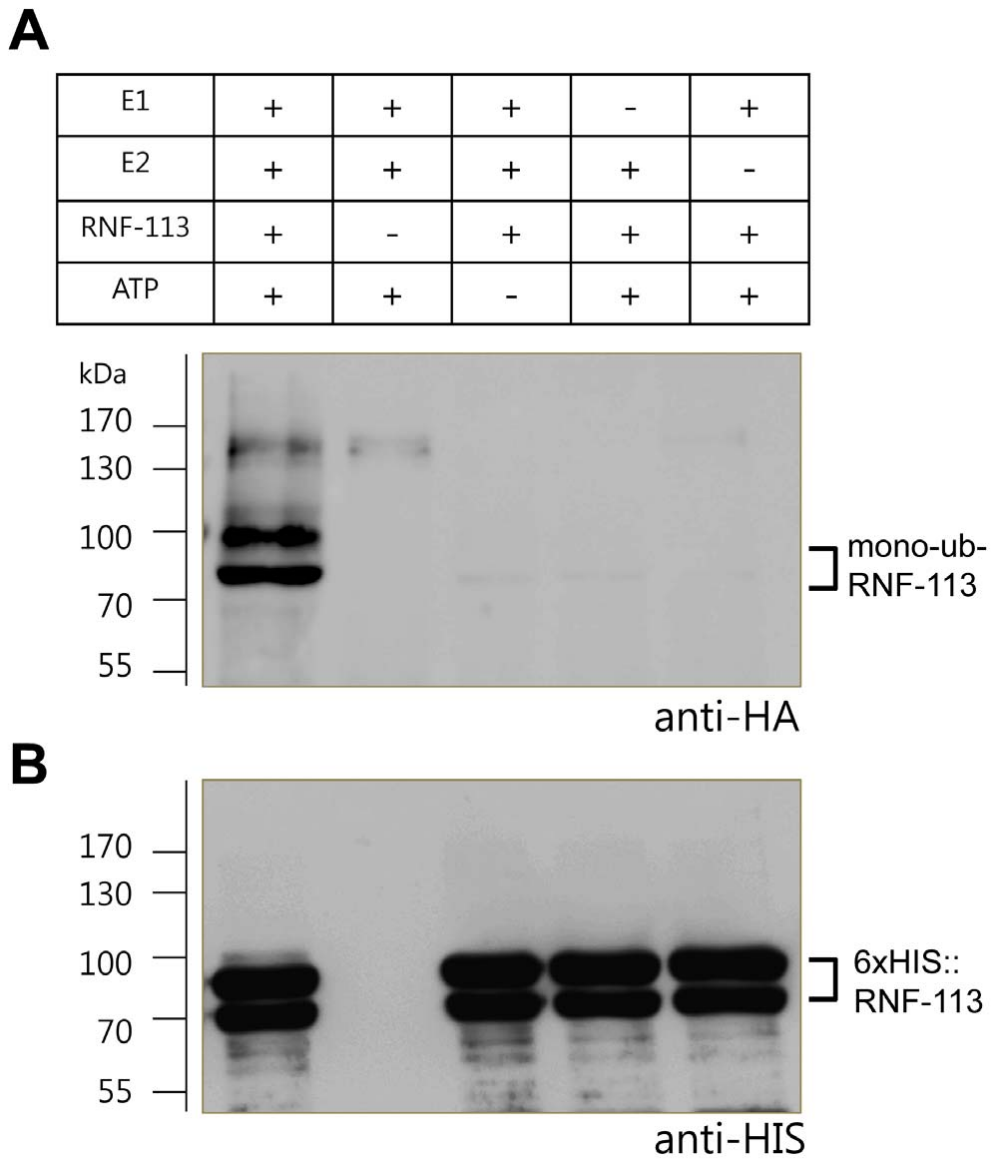
RNF-113 has E3 ubiquitin ligase activity in vitro. Recombinant 6×HIS::RNF-113 was purified from *E. coli* lysates using Ni-NTA agarose and incubated with E1, HA-ubiquitin, and UbcH5c as E2 enzyme. The reaction products were electrophoresed in two separate 6–18% SDS-polyacrylamide gels and probed with (A) HA and (B) HIS antibodies, respectively.

To understand the molecular function of RNF-113, we measured its enzymatic activity by expressing it in *E. coli* with an N-terminal 6×HIS tag and purifying the tagged protein on a Ni-NTA column. The purified protein was reacted with E2 (UbcH5c) in the presence of E1, ubiquitin (HA-tagged), and ATP. The reaction products were separated on an 8–16% SDS-polyacrylamide gel and analyzed by western blotting using HA antibody. When all the components for ubiquitination, namely HA-ubiquitin, E1, E2, RNF-113, and ATP were present, two ubiquitinated protein bands of about 100 kDa and 80 kDa were formed (Figure 5A). The bands were absent if ATP, E1 or E2 was omitted. To characterize the two ubiquitinated proteins, the reaction products of Figure 5A were separated on another 8–16% SDS-PAGE and probed with HIS antibody. Only the lanes containing RNF-113 exhibited two His-tagged proteins of about 90 kDa and 70 kDa (Figure 5B), which were also observed after protein-staining with Ponceau S (data not shown). Although the reason why two HIS-tagged polypeptides were produced is not clear (GST-tagged RNF-113 used for antibody production was also expressed as two polypeptides), both polypeptides were confirmed to be RNF-113 by MALDI-TOF mass spectrometric analysis (data not shown). To show that the ubiquitinated proteins (Figure 5A) are derived from the two HIS-tagged proteins, we analyzed the ubiquitinated proteins by two-dimensional electrophoresis involving isoelectric focusing followed by SDS-PAGE (Figure S6 File S1). The longer polypeptide having the expected isoelectric point is thought to be the full-length 6×HIS::RNF-113, whereas the shorter one with a higher isoelectric point is probably a cleaved product. The results (Figures 5 and S6 in File S1) are most consistent with the argument that the two ubiquitinated bands (Figure 5A) correspond to mono-ubiquitinated forms of the two 6×HIS::RNF-113 polypeptides (Figure 5B).

## Discussion

In this study, we have shown that the *C. elegans* ring finger protein, RNF-113, does not affect FCD-2 (FANCD2 ortholog) focus formation, thus excluding its possible role as a ubiquitin ligase for FCD-2 (Figure 2). However, the protein has been shown to be a regulator of RAD-51 focus formation in response to interstrand DNA crosslinks (ICLs) (Figures 3A and 3C). The purified protein had E3 ligase activity, adding mono-ubiquitin to itself in the presence of E1, E2, and ATP (Figure 5). How this E3 activity is related to its role as a regulator of RAD-51 focus formation after ICL treatment is unclear. One clue to the molecular function of RNF-113 is its epistatic interaction with RFS-1, a RAD51C homolog. RFS-1 is required for the full effects of RNF-113 depletion on embryonic lethality and brood size in untreated worms, and for persistence of RPA-1 foci in the germ cells after ICL treatment (Figures 1A, S2A in File S1, and 4). The mammalian homolog of RFS-1, RAD51C, is thought to play a role at more than one stage of homologous recombination, including RAD51 filament formation and the subsequent Holliday junction formation [15], [16], [46]. Nevertheless, *C. elegans* RFS-1 is more important for RAD-51 focus formation after ICL formation than after treatment with ionizing radiation [28]. Likewise, the role of mammalian RAD51C may be more critical in ICL repair than in DSB repair [1].

**Figure 6:**
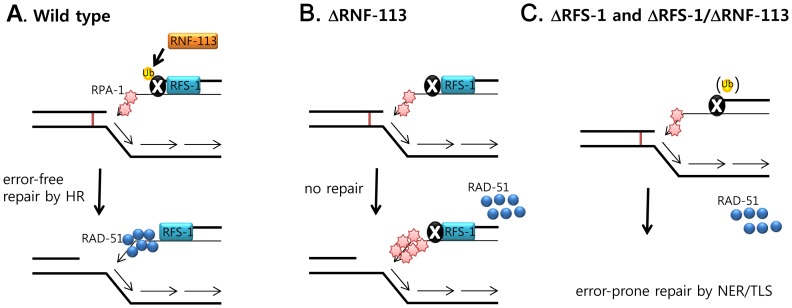
Proposed model on the roles of RNF-113 and RFS-1 in loading RAD-51 to replication forks stalled at ICLs. (A) In the wild-type background, a replication fork stalls spontaneously at an ICL and is cleaved. The resulting one-ended DSB is resected to produce ssDNA (single-strand DNA), to which RPA-1, RFS-1 and an unknown protein ‘X’ bind. RNF-113 is proposed to ubiquitinate X, and the ubiquitinated X together with RFS-1 promotes replacement of RPA-1 on ssDNA by RAD-51. (B) In the absence of RNF-113, X is not ubiquitinated so that RPA-1 cannot be displaced from ssDNA. The presence of RPA-1, together with RFS-1 and unmodified X on ssDNA prevents the loading of RAD-51, and the DNA intermediate is at a dead end. (C) In the absence of RFS-1, RAD-51 cannot be recruited to ssRNA, even though RNF-113 ubiquitinates X on ssDNA. Nevertheless, the DNA intermediate with RPA-1 and ubiquitinated X bound is shunted to an error-prone repair pathway involving nucleotide excision repair (NER) and translesion DNA synthesis (TLS). In the absence of both RNF-113 and RFS-1, the DNA intermediate with RPA-1 and unmodified X is also shunted to the error-prone repair pathway.

In view of the known roles of RFS-1/RAD51C, and also the effects of RNF-113 on the dissipation of ICL-induced RPA-1 foci, RNF-113 appears to replace, probably indirectly, RPA-1 at DNA replication forks stalled by ICLs (Figure 6A). Stalled replication forks are predominantly processed by DNA incision to yield one-ended DSBs, which are then repaired by homologous recombination [47]. We propose that RFS-1 binds to single-stranded DNA (ssDNA) derived from one-ended DSBs by end-resection, like mammalian RAD51C, which forms nuclear foci concomitantly with RAD51 and RPA at DSBs [48]; thereafter RNF-113 ubiquitinates an unknown protein ‘X’ that could be RFS-1 or a subunit of RPA, at which point RPA-1 dissociates from the DNA (Figure 6A). Thereafter, RAD-51 can be loaded onto the ssDNA, and replication fork recovery is initiated via strand invasion during homologous recombination. In the absence of RNF-113 the nuclear protein ‘X’ cannot be ubiquitinated; hence RPA-1 remains associated with the ssDNA, preventing loading of RAD-51 (Figure 6B). The persistence of RPA-1 together with RFS-1 and ‘X’ on the ssDNA probably inhibits further processing of the fork by either homologous recombination or by an alternative repair pathway. In the absence RFS-1 (Figure 6C), RAD-51 cannot be recruited to the ssDNA, and the DNA intermediate with RPA-1 and ‘X’ bound to it, is processed by an error-prone repair pathway, probably involving nucleotide excision repair and translesion DNA synthesis. The situations in Figure 6C contrasts with the case in Figure 6B (RNF-113 single deficiency) where the DNA intermediate with RFS-1, RPA-1 and ‘X’ all bound, is committed to homologous recombination but frozen at that step.

Although we have discussed the roles of RNF-113 in relation to DNA damage repair, we noted that RNF-113 was important for embryonic survival in the absence of exogenous DNA insults. Likewise, homozygotes of a deletion allele *rnf-113*(*ok1401*) have been reported in WormBase (http://www.wormbase.org/) to arrest at mid-larval stages. Therefore, RNF-113 appears to be essential for larval growth as well as for embryogenesis. Accordingly, the protein has been reported as an enhancer of the tumorous germ line proliferation of *glp-1(oz64)* [49]. Knockdown of a number of proteins participating in pre-mRNA splicing induced the same phenotype, supporting a function of RNF-113 in pre-mRNA splicing. Additional support for a role of RNF-113 in pre-mRNA splicing comes from the fact that its homolog CWC24p in *S. cerevisiae* is involved in splicing of pre-U3 snoRNA, therefore affecting pre-rRNA processing [50]. CWC24p has also been identified as a component of Cef1p complexes involved in pre-mRNA splicing, which also contain PRP19 [51], [52]. It is intriguing that *S. cerevisiae* mutated in PRP19/PSO4 is hypersensitive to DNA damage, as is *C. elegans* deficient in RNF-113 [53]. Given the role of RNF-113 in promoting tumorous germ cell proliferation like other splicing factors, and the activity of its homolog in *S. cerevisiae* on pre-U3 snoRNA splicing, it is very possible that RNF-113 participates in pre-RNA splicing. What we observed after knocking down RNF-113 expression could theoretically have been an indirect effect of inhibiting the transcription of ICL repair genes. However, the embryonic lethality and persistent RPA-1 foci due to *rnf-113* depletion were not induced in the absence of RFS-1, thus supporting a direct link between the two proteins.

In summary, our study has identified a novel ubiquitin ligase RNF-113 in cellular response to interstrand DNA crosslinks in *C. elegans*. The protein promotes RAD-51 focus formation and shows genetic interactions with the RAD51C ortholog, RFS-1. It will be important to identify target proteins ubiquitinated by RNF-113 and to find out whether RFS-1 or a subunit of RPA complex is one of the targets in vivo, to fully elucidate the biological functions of RNF-113.

## Materials and Methods

### *C. elegans* Strains

Bristol N2 *C. elegans* worms were cultured at 20°C on agar containing nematode growth medium seeded with *E. coli* OP50-1 cells. Nematode mutations used were *fcd-2(tm1298)* and *rfs-1(ok1372)*. The mutation *fcd-2(tm1298)* was generated by the National Bioresource Project (Japan) and previously out-crossed 6 times with wild-type N2 males [27]. The *rfs-1(ok1372)* mutant was obtained from the *Caenorhabditis* Genetics Center and out-crossed to the wild-type N2 strain 3 times.

### RNA interference

Wild-type N2 or mutant worms were fed *E. coli* strain HT115(DE3) that had been transformed with the L4440 feeding vector (control), or the same vector carrying an appropriate cDNA insert. The full length cDNA of *rnf-113* was amplified from a *C. elegans* cDNA pool using primers: 5′-GGATCCATGGATCTCTTCCGAAAAC and 5′-AAGCTTTCAATCTTTTTCAGCATCAT (restriction sites underlined). Knockdown of *chk-1* was performed using the feeding vector described in our previous work [54]. Since *rnf-113* is co-transcribed at its 3′-end with *hpl-2*, which is a heterochromatin protein HP1 homolog [55], we checked whether *hpl-2* mRNA expression was affected by the knockdown of *rnf-113*. The level of *hpl-2* mRNA was not affected by the RNA*i*; hence the hypersensitivity of *rnf-113*(RNA*i*) worms is only due to loss of RNF-113, not to any effects on HPL-2 (Figure S4B in File S1). The fact that *rnf-113*(RNA*i*) worms were not hypersensitivity to γ-rays (Figures 3B and S5B in File S1), unlike a *hpl-2* mutant that showed a great hypersensitivity to the radiation [55], also supports that *hpl-2* expression was not significantly affected by the knockdown.

### Embryonic lethality after ICL treatment

Synchronized L1 worms were transferred to NGM plates that had been seeded with *E. coli* HT115(DE3) cells in the presence of 1 mM IPTG. The *E. coli* strain HT115(DE3) contained either the L4440 control vector or the vector with a cDNA insert. When worms reached the late L4 stage at 20°C, they were soaked in 1× PTW containing 25 µg/ml TMP (4,5′,8-trimethylpsoralen, Sigma) for 30 min, and irradiated with 200 J/m^2^ of ultraviolet light (365 nm). Ten of the treated worms were individually placed on an NGM plate with *E. coli* cells and allowed to lay eggs for 24 h. Hatched eggs were scored 20 h later to calculate hatching rate. All experiments were done in triplicate.

### Antibody preparation

The full length cDNA of RNF-113 was amplified from a cDNA pool that had been prepared by reverse transcription of total RNA from wild-type *C. elegans*. Primers were 5′-GGATCCATGGATCTCTTCCGAAAAC and 5′-CTCGAGTCAATCTTTTTCAGCATCATC, and the amplified DNA fragment was inserted between the *Bam*HI and *Xho*I sites on pGEX4T-1 and transformed into BL21(DE3) cells (Yeastern Biotech). The recombinant protein was overexpressed by incubation with 1 mM IPTG for 4 h at 37°C, and used to generate antibodies in rats. The polyclonal antibody against RNF-113 was isolated from the serum of the immunized rats by affinity chromatography as follows. The GST::RNF-113 protein was blotted onto a nitrocellulose membrane, and the membrane was incubated with the antiserum. The antibody was the stripped off the membrane in 100 mM glycine (pH 2.5), and the resulting antibody solution was neutralized and concentrated to be used for immunostaining.

### Immunostaining

*C. elegans* gonads were ejected from the body with a scalpel, and fixed in 4% paraformaldehyde followed by 100% methanol. They were then incubated with RAD-51(1∶50 dilution), RPA-1(1∶1,000 dilution), FCD-2(1∶25 dilution), or RNF-113(1∶50 dilution) antibodies for 16 h at 4°C [33]. The gonads were reacted with Alexa Fluor 488-conjugated IgG anti-rat antibody (Molecular probes, 1∶1,000), stained with DAPI (4,6-diamidino-2-phenylindole, 1 mg/ml), and observed with a fluorescence microscope (DMR HC, Leica).

### In vitro ubiquitination assay

The full length cDNA of RNF-113, amplified as above, was inserted between the *Bam*HI and *Xho*I sites of pET-32a (Novagen) for overexpression of 6×HIS::RNF-113. BL21(DE3) cells were transformed with the recombinant plasmid DNA, and overexpression was induced in the presence of 1 mM IPTG at 37°C for 3 h. The recombinant protein was purified from *E. coli* lysates using Ni-NTA agarose (QIAGEN).

In vitro ubiquitination by RNF-113 was assayed using a modification of the protocol of Alpi et al. [56]. The reaction mixture (10 µl) contained 0.4 µM E1, 3 µM E2 (UbcH5a), 25 mM HA-ubiquitin, 5 mM ATP-Mg^2+^, and 1 µg 6×HIS::RNF-113 in 1× PBS buffer. E1, E2, HA-ubiquitin, and ATP-Mg^2+^ were purchased from Boston Biochem. After incubation at 37°C for 60 min, the reaction was stopped by adding reducing SDS sample buffer and boiling for 5 min. The reaction products were separated by 6–18% pore-gradient SDS-PAGE, transferred to nitrocellulose membrane, and blotted with anti-HA or anti-HIS antibody (Sigma, 1∶5000 dilution).

### Western blot analysis

About 600 wild-type worms at the L4 or young adult stage were collected after knocking down *rnf-113* expression from the L1 stage. They were soaked in 25 µg/ml TMP (4,5′,8-trimethylpsoralen) for 30 minutes and then exposed to UVA (365 nm, 200 J/m^2^). The worms were grown further for 16 h on NGM plates seeded with *E. coli*, collected, and boiled in reducing SDS sample buffer. Proteins were separated by 10% SDS-PAGE and transferred onto a nitrocellulose membrane. Anti-RAD-51 rat antiserum (1∶1,000) and anti-α-tubulin monoclonal mouse antibody (1∶5,000) were used as primary antibodies, followed by anti-rat and anti-mouse HRP antibodies (Santa Cruz Biotechnology) as secondary antibodies. Electrochemical luminescence assays were performed using WESTSAVEUp (AbFRONTIER). Luminescence signals were detected with a LAS-3000 imaging system (Fujifilm).

### Two-dimensional gel electrophoresis

The reaction products of an in vitro ubiquitination (50 µl) involving 6×HIS::RNF-113, HA-ubiquitin, E1, and E2, were concentrated using Vivaspin (Satorius, Germany) in the buffer (9 M Urea, 4% CHAPS). After adding the rehydration buffer (IPG buffer, 5 mM DTT, bromophenol blue), the reaction products were applied to an IPG strip (pH 4–7, 7 cm, GE Healthcare). After 16 h of rehydration at room temperature, the IPG strip was placed into the Ettan IPG phor 3 system (GE Healthcare) to perform isoelectric focusing. The applied voltage was increased gradually from 100 V to 3500 V for 9.6 h. After isoelectric focusing, the strip was equilibrated with the buffer (50 mM Tris⋅Cl, pH 8.8, 6 M urea, 20% glycerol, 2% SDS, 2.5% acrylamide, 0.54% tributylphosphine) and placed on an 8–16% SDS-PAGE. After second-dimensional gel electrophoresis, proteins were transferred to a nitrocellulose membrane and detected using HIS or HA antibody (Sigma, 1∶5000).

## Supporting Information

File S1 **Figure S1.** Comparison of amino acid sequences of Ring finger protein 113 homologs in *Homo sapiens* and *C. elegans*. Both proteins have a zinc finger domain (yellow) and a ring finger domain (red), and are identical in 35% of their amino acids. Alignment was carried out with Vector NTI Advance (10.0.1) software. **Figure S2.** Differential effects of RNF-113 depletion on brood size and oocyte chromosomal abnormalities in wild type, *fcd-2*, and *rfs-1* backgrounds. (A) Knockdown of *rnf-113* was performed by feeding RNA*i* from the young adult stage (P0 generation) of wild-type, *fcd-2(tm1298)*, and *rfs-1(ok1372)* worms. The total numbers of eggs laid during the first 3 days of F1 adults were determined. (B) The gonads of F1 adult worms (n = 20) were dissected and stained with DAPI, to count endomitotic (Emo) oocytes. The error bars are SEM. The scale bar is 10 µm. **Figure S3.** Effects of RNF-113 depletion on RAD-51 focus formation with time lapse after ICL treatment. (A) The mitotically proliferating regions of gonads from wild-type and *rnf-113*(RNA*i*) worms are shown after staining for RAD-51 at 9 and 18 h following TMP/UVA treatment. (B) The immuno-staining in (A) was repeated at 3 and 9 h after IR (ionizing radiation, 75 Gy) treatment instead of ICL treatment. The scale bars are 10 µm. **Figure S4.** Knockdown of *rnf-113* expression does not affect the level of RAD-51 protein or *hpl-2* transcripts. (A) Western blot of extracts of wild type, *rnf-113*(RNA*i*), and *chk-1*(RNA*i*) worms at the adult stage using antibodies to RAD-51 and α-tubulin. The upper band of RAD-51 is thought to be its phosphorylated form, pRAD-51. (B) *hpl-2* expression relative to that of γ-tubulin. Mixed stages of wild type and *rnf-113*(RNA*i*) worms were harvested 18 h after ICL treatment and total RNA isolated. Briefly, 2 µg of total RNA was used to synthesize a strand of cDNA using oligo(dT) primer and AMV reverse transcriptase (Intron, Korea). The resulting cDNA was amplified using iQ SYBR Green Supermix (Bio-Rad) in a real time PCR instrument (CFX96 Touch, Bio-Rad). cDNA amplification was analyzed with CFX Manager Software. The primers were 5′-GGACGAGTTTGAGAGGGAA and 5′-CTGCTTGCCTTCCAGTGA for *hpl-2*, and 5′-AAGATCTATTGTTCTACCAGGC and 5′-CTTGAACTTCTTGTCCTTGAC for γ-tubulin. **Figure S5.** Effects of ionizing radiation (IR) on the intracellular location of RNF-113 and on embryonic survival after RNF-113 knockdown. (A) Intracellular localization of RNF-113 in the germ cells of the mitotically proliferating region of wild-type *C. elegans* gonads 3 h after γ-ray (75 Gy) treatment. (B) Hatching rate of embryos derived from germ cells that had been treated with γ-rays (75 Gy) is not affected by RNF-113 depletion. **Figure S6.** Analysis of two forms of 6×HIS::RNF-113 that were ubiquitinated in vitro by two-dimensional gel electrophoresis. Isoelectric focusing (pH 4–7) was followed by 8–16% SDS-PAGE, and only the left part (corresponding to pH 4–6) of a gel is shown. (A) Detection of 6×HIS::RNF-113 before in vitro ubiquitination using HIS antibody. (B) 6×HIS::RNF-113 was reacted with HA-ubiquitin, E1, E2, and ATP, and the reaction products were detected using HA antibody, deprobed, and then reprobed with HIS antibody.
(PDF)
