# Supplementary figures and images for: C. elegans Ring Finger Protein RNF-113 Is Involved in Interstrand DNA Crosslink Repair and Interacts with a RAD51C Homolog
Source: PLoS One. 2013 Mar 28;8(3):e60071. doi: 10.1371/journal.pone.0060071 (PMC3610817; doi:10.1371/journal.pone.0060071)

Fig. S1

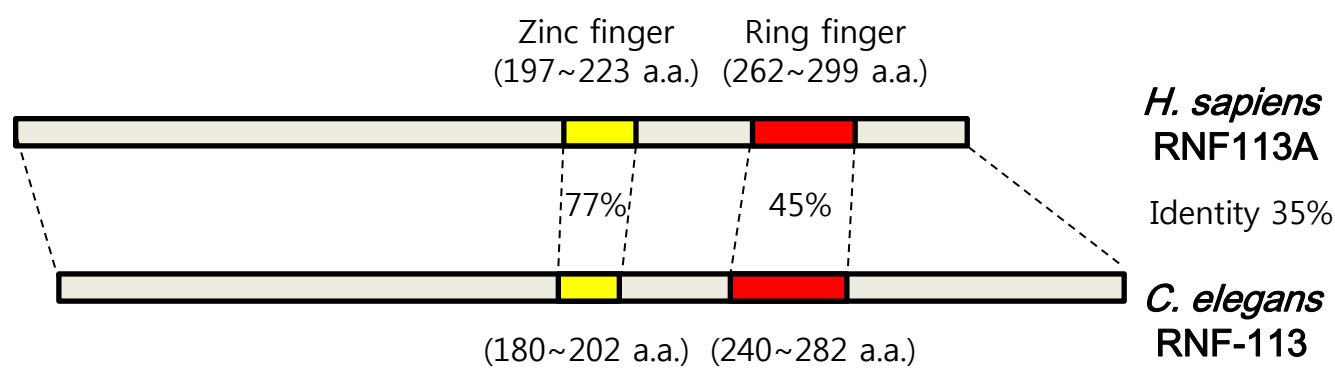

**Fig. S2**

**A**

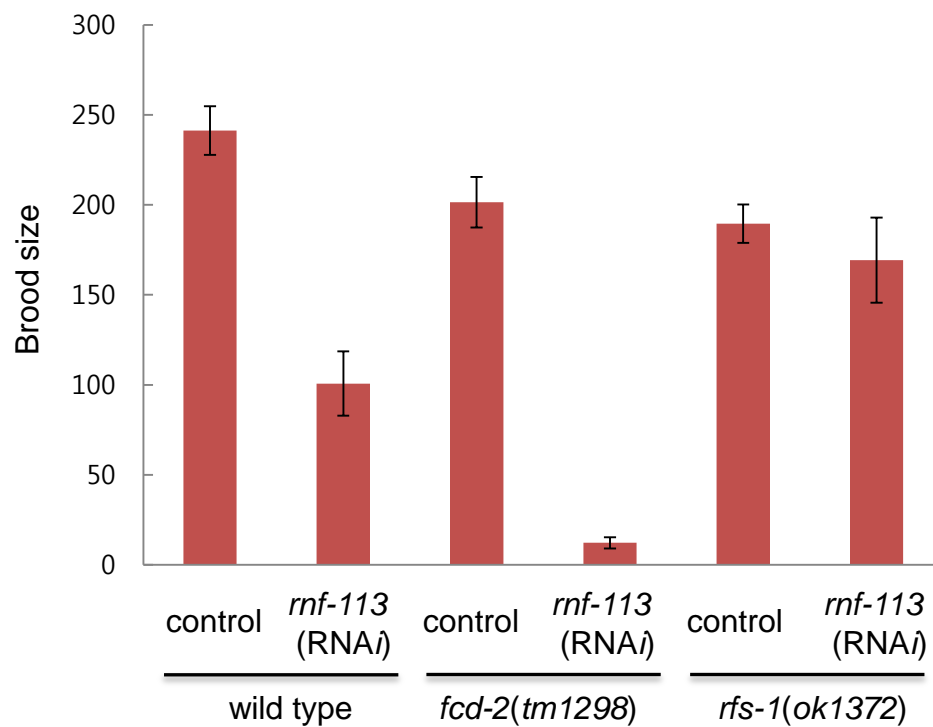

**B**

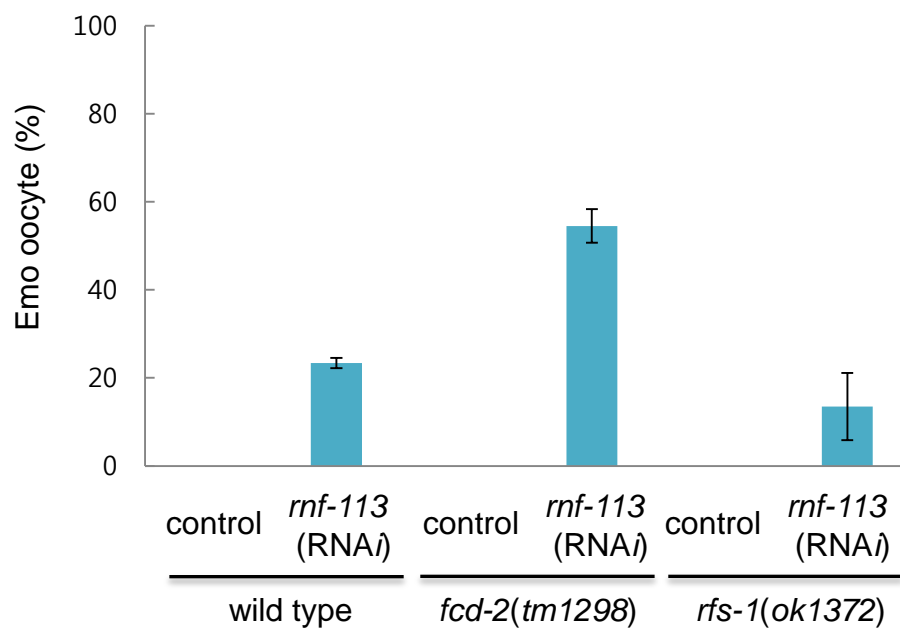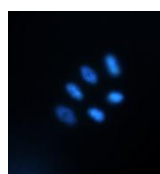

normal oocyte

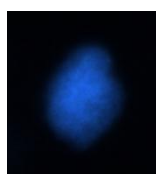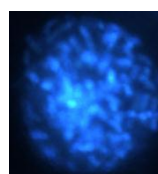

Emo oocytes

**Fig. S3**

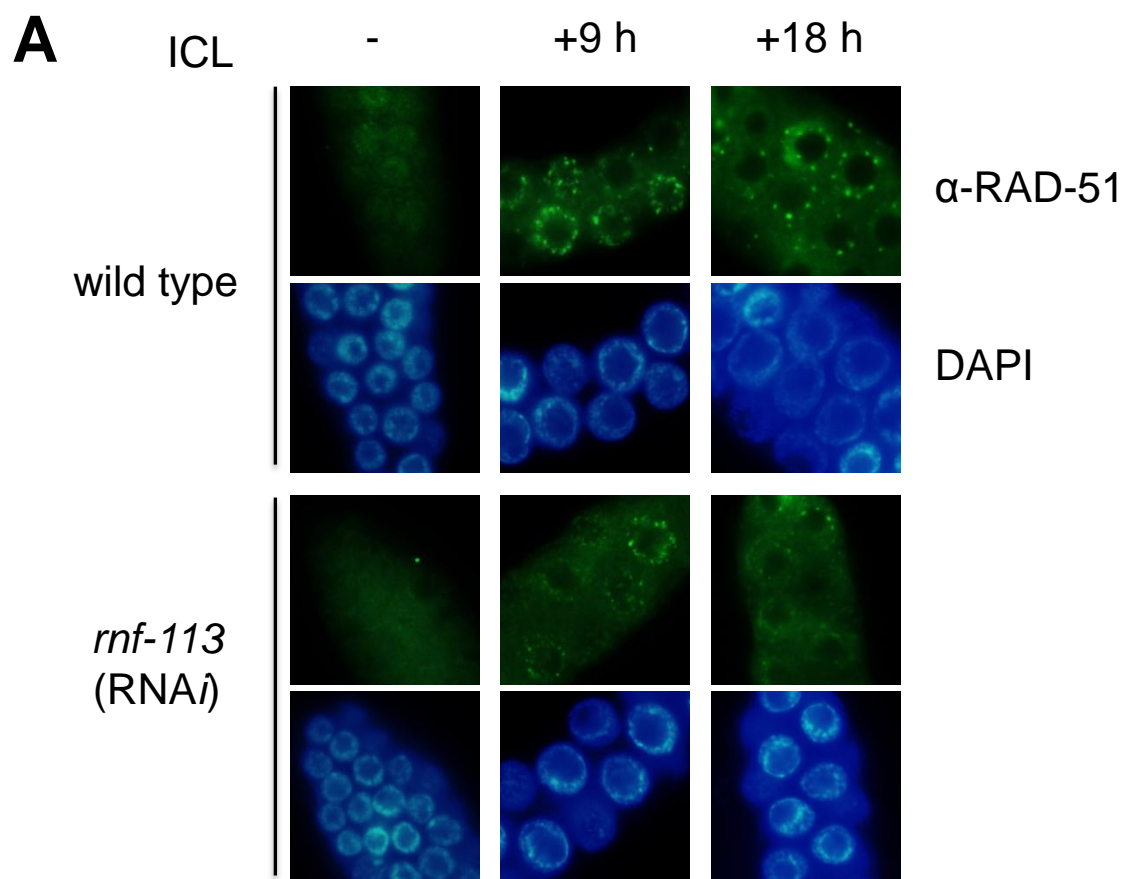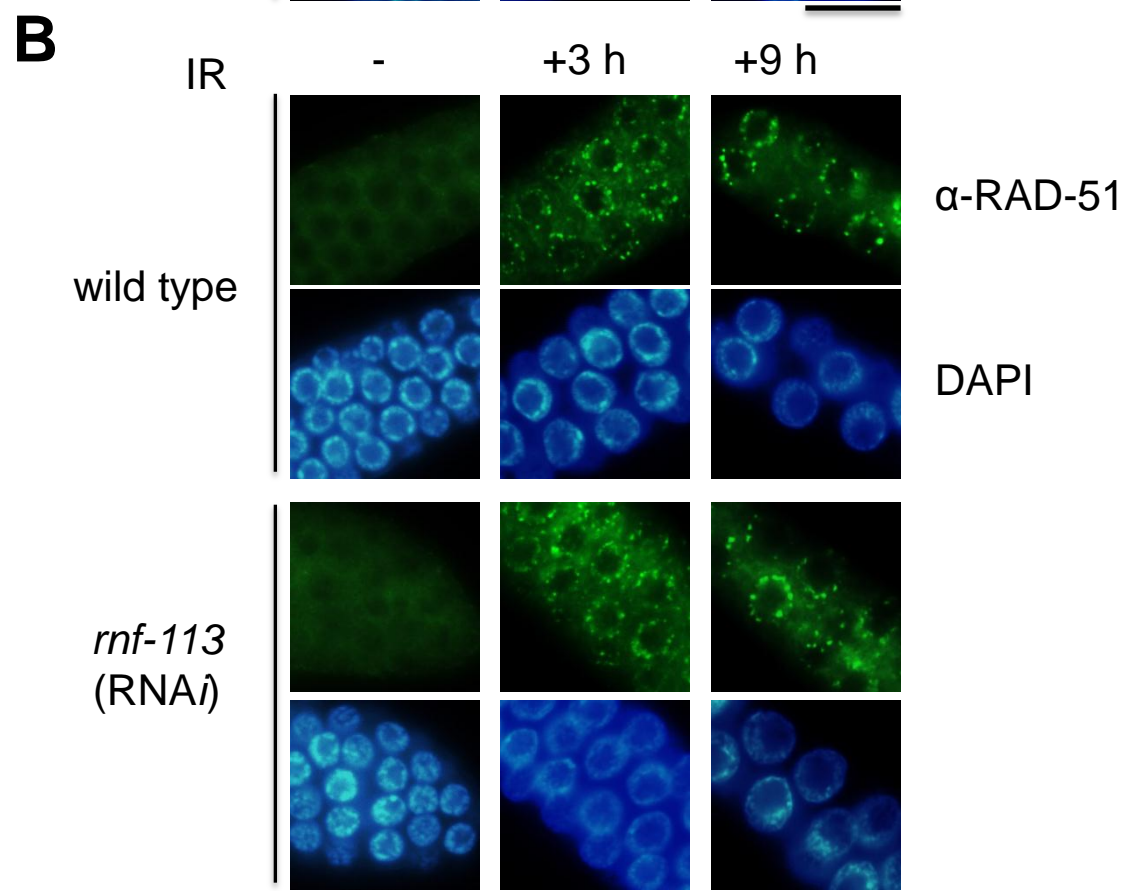

**Fig. S4**

**A**

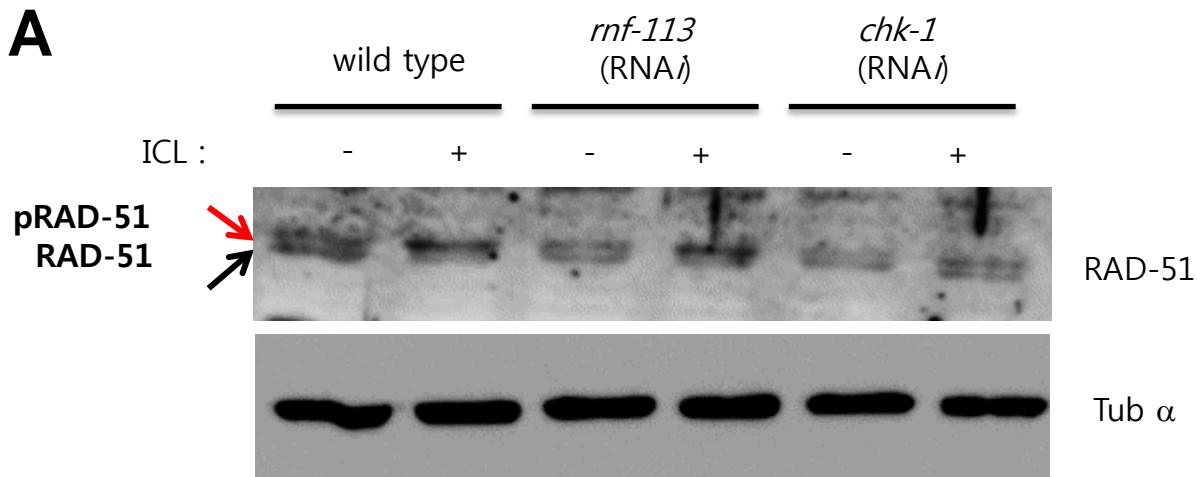

**B**

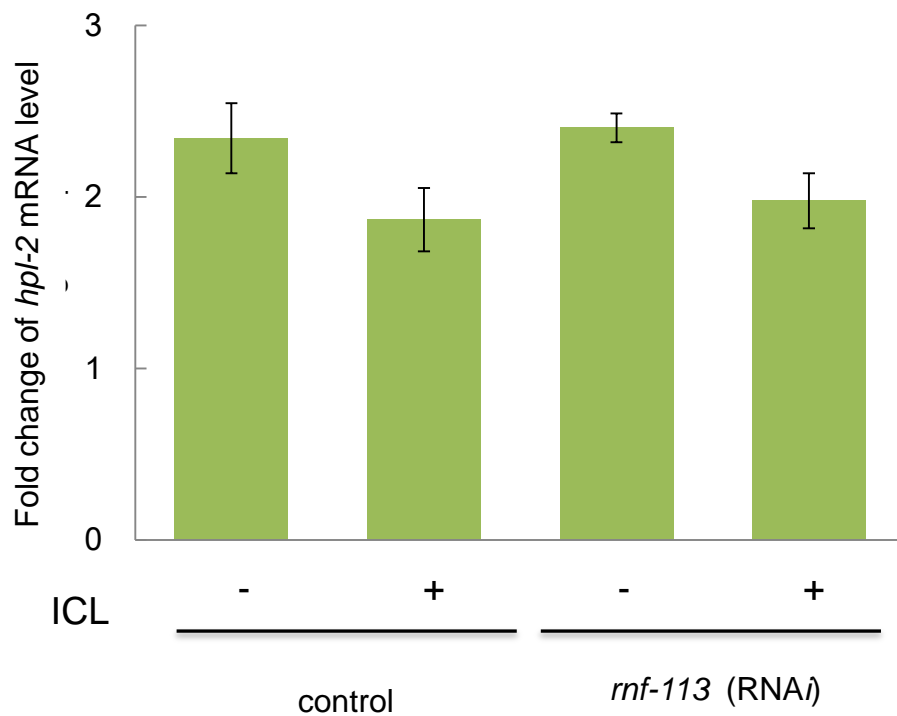

**Fig. S5**

**A**

wild type

IR

—

 $+5h$ 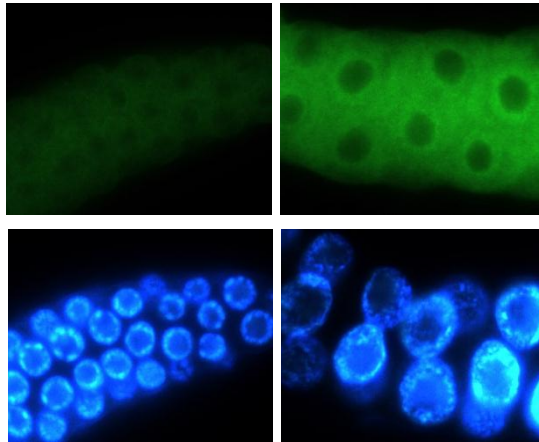

**B**

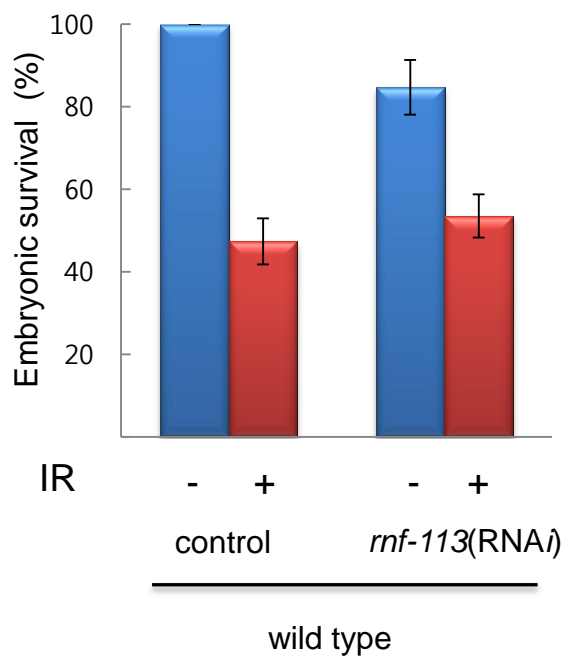

**Fig. S6**

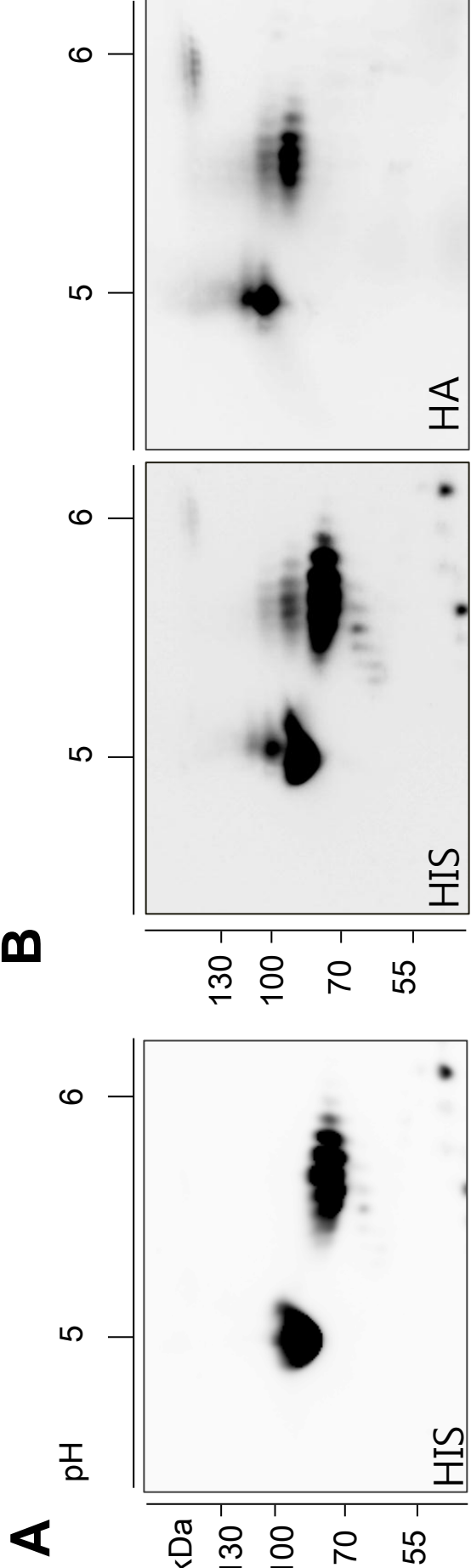

Supplement: File S1 — Figure S1. Comparison of amino acid sequences of Ring finger protein 113 homologs in Homo sapiens and C. elegans. Both proteins have a zinc finger domain (yellow) and a ring finger domain (red), and are identical in 35% of their amino acids. Alignment was carried out with Vector NTI Advance (10.0.1) software. Figure S2. Differential effects of RNF-113 depletion on brood size and oocyte chromosomal abnormalities in wild type, fcd-2, and rfs-1 backgrounds. (A) Knockdown of rnf-113 was performed by feeding RNAi from the young adult stage (P0 generation) of wild-type, fcd-2(tm1298), and rfs-1(ok1372) worms. The total numbers of eggs laid during the first 3 days of F1 adults were determined. (B) The gonads of F1 adult worms (n = 20) were dissected and stained with DAPI, to count endomitotic (Emo) oocytes. The error bars are SEM. The scale bar is 10 µm. Figure S3. Effects of RNF-113 depletion on RAD-51 focus formation with time lapse after ICL treatment. (A) The mitotically proliferating regions of gonads from wild-type and rnf-113(RNAi) worms are shown after staining for RAD-51 at 9 and 18 h following TMP/UVA treatment. (B) The immuno-staining in (A) was repeated at 3 and 9 h after IR (ionizing radiation, 75 Gy) treatment instead of ICL treatment. The scale bars are 10 µm. Figure S4. Knockdown of rnf-113 expression does not affect the level of RAD-51 protein or hpl-2 transcripts. (A) Western blot of extracts of wild type, rnf-113(RNAi), and chk-1(RNAi) worms at the adult stage using antibodies to RAD-51 and α-tubulin. The upper band of RAD-51 is thought to be its phosphorylated form, pRAD-51. (B) hpl-2 expression relative to that of γ-tubulin. Mixed stages of wild type and rnf-113(RNAi) worms were harvested 18 h after ICL treatment and total RNA isolated. Briefly, 2 µg of total RNA was used to synthesize a strand of cDNA using oligo(dT) primer and AMV reverse transcriptase (Intron, Korea). The resulting cDNA was amplified using iQ SYBR Green Supermix (Bio-Rad) in a real [file pone.0060071.s001.pdf]
